# COVID-19 infection prevention and control procedures and institutional trust: Perceptions of Palestinian healthcare workers

**DOI:** 10.3389/fpubh.2022.947593

**Published:** 2022-08-19

**Authors:** Nuha El Sharif, Muna Ahmead, Asma Imam

**Affiliations:** Faculty of Public Health, Al Quds University, Jerusalem, Palestine

**Keywords:** perception, healthcare workers, institutional trust, COVID-19, Palestine

## Abstract

**Background:**

Lack of trust in institutional control measures during Coronavirus disease 2019 (COVID-19) outbreaks may affect healthcare workers' (HCWs) levels of stress and wellbeing, and as a consequence, may influence their trust and confidence in their organization. This study aims to understand factors associated with healthcare workers perceptions of trust in organizational preparedness, communication, and infection risk during the COVID-19 pandemic.

**Methods:**

A cross-sectional study was conducted among HCWs (*n* = 876) in 16 COVID 19 healthcare facilities between October and December 2020 in Palestine (Gaza Strip, West Bank and East Jerusalem). A stratified purposive sample using an online self-administered Arabic version of a questionnaire was used for data collection. The questionnaire used for this study was adapted from the World Health Organization Blueprint Novel Coronavirus Perceptions of healthcare workers regarding local infection prevention and control procedures for a COVID-19 research protocol.

Data were analyzed using Statistical Package for Social Sciences software version 23. In the bivariate analysis, *T*-test, one-way ANOVA and χ2 test were used at a significant *p*-value < 0.05. In the multivariable logistic regression analyses, the adjusted odds ratios and its 95% confidence intervals are presented.

**Results:**

Findings showed that confidence in the systems' ability to manage COVID-19 cases, encouragement and support from senior medical/nursing staff to apply recommended IPC measures, and good levels of mental health increased trust in the organization. Additionally, receiving proper training on IPC procedures for other communicable diseases, having access to clear policies and procedures related to IPC procedures for COVID-19, and providing PPE during the previous clinical shift also increased trust. However, the intention to use recommended PPE when treating patients with suspected or confirmed COVID-19 when having access to it and feeling emotional was negatively correlated with this trust.

**Conclusions:**

HCWs should be provided with clear, accessible communications about policies and protocols, as well as training about infection prevention and control, personal protective equipment, and support during pandemics to increase their trust in the healthcare system. Additionally, the improvement in HCWs' wellbeing can be attributed to a greater sense of trust in institutions.

## Background

The Coronavirus disease 2019 (COVID-19) pandemic has posed exceptional challenges and threats to healthcare systems globally with millions of confirmed cases and deaths (1). The pandemic has had a major impact on the capacity of health systems to continue the delivery of essential health services and has put intense pressure on healthcare workers (HCWs) and resources (2). Frontline HCWs are at a higher risk of infection and death due to their direct contact with COVID-19 patients; the pandemic has caused the deaths of more healthcare workers than any other disease outbreak (3), in addition to the effect on their physical and mental health (4).

The literature underscores the importance of healthcare workers' trust and psychological safety as pre-requisites for organizational resilience in healthcare organizations (5–7). Resilience depends on several factors such as planning, perception, organizational trust and reaction to unexpected conditions such as a pandemic (8). The COVID-19 pandemic highlights the importance of organizational trust for healthcare workers to make tradeoffs, communicate safety concerns to managers and improve organizational resilience. The absence of leadership support for HCWs during the COVID-19 pandemic was suggested as a factor in emotional distress and burnout (6). Ultimately, lack of support may undermine the trust needed for healthcare workers to communicate patient safety concerns to their managers (7). In addition, lack of confidence and trust in institutional control measures during COVID-19 outbreaks may have an impact on HCWs levels of stress and subjective wellbeing, including cognitive and emotional dimensions such as anxiety, worry, fear, sadness and tearfulness (2). This may influence HCWs perceptions and confidence in carrying out and adhering to infection prevention and control (IPC) procedures (4, 9–12), and could increase their risk of becoming infected (13). Thus, lack of trust has a substantial effect on the physical and mental health of HCWs, and the quality of care delivered to patients within clinical settings (12, 14).

Previous studies showed poor compliance of healthcare workers with infection prevention and control (IPC) measures in practice (15, 16), which are crucial to preventing the spread of infection caused by COVID-19 (15). Therefore, HCWs should apply appropriate IPC behaviors including personal protective equipment (PPE) use and hand hygiene, to protect patients and themselves from infection (14–16). In China, Wuhan (2021), HCWs reported good IPC behaviors, while the compliance with goggle and gown use was relatively low (below 85%). In terms of hand hygiene and droplet isolation behaviors, environmental context and resources domain were significantly correlated. Environmental context, knowledge domain and emotion domain were all significantly related to goggle and gown use. Overall droplet isolation behaviors and gown use were also predicted by social influences (17).

In the COVID-19 pandemic, personal protective equipment (PPE) usage and trust in institutions' differing recommendations and requirements have become major concerns. Protection for HCWs by providing personal protective equipment (PPE), training, addressing fatigue, and treating the psychosocial consequences of the outbreak are seen as a crucial task of health organizations globally and are measures linked to institutional trust (18–21). Therefore, the health organization must ensure the provision of medical supplies based on need, type, quality and quantity, in addition to appropriate psychological support, interventions and staff support measures.

Limited number of studies was done on trusting organization during COVID-19. A study in Nigeria showed that a significant relationship between trust in the health facility and the provision of clear accessible policies and protocols regarding IPC, personal protective equipment and support (22). Another study in Canada showed that nurses without experience working in outbreak settings had higher levels of fear of becoming ill and fear of providing care for COVID-19 patients compared to the experienced nurses who had better Infection Prevention and Control (IPC) skills and easier access to personal protective equipment (23). In a study, health workers in India reported physical fatigue, dehydration, weight loss, suffocation, rash eruptions, and exhaustion due to increased work hours and the use of personal protective equipment kits. In addition, due to their fear of infection and their increased workload, HCWs reported being socially isolated from friends and family (24). A local Palestinian study showed that fear of COVID-19 was positively correlated with depression, anxiety and stress among psychosocial service providers. In addition, fear of COVID-19 and psychological distress was fully mediated by wellbeing (25).

In the Occupied Palestinian Territories, as in other lower-middle income countries dealing with conflict (26), the resources available to deal with COVID-19 were (and are still) scarce and there was no emergency plan to deal with such a scenario. District emergency committees were activated across all governorates in preparedness, and training targeted medical and non-medical personnel working in primary, secondary and emergency health services. Therefore, this study aims to understand factors associated with healthcare workers perceptions of trust in organizational preparedness, communication, and infection risk during the COVID-19 pandemic.

## Materials and methods

### Study design

A descriptive cross-sectional survey was conducted among healthcare workers during the period of October to December 2020.

### Study settings and sampling

The study was implemented in the West Bank, Gaza Strip, and East Jerusalem. The Palestinian Authority and the authority in the Gaza Strip assume responsibilities for administration of public health-care provision to the Palestinian population. The Palestinian health care system faces barriers in the form of permit restrictions that limit Palestinian access to health care. Restrictions on access and movement are common in Palestine, and they make access to health care incredibly difficult. In addition, in East Jerusalem, six Palestinian hospitals are the main providers of tertiary referral care for Palestinians in the West Bank and Gaza Strip for health services of which the Ministry of Health is unable to provide. But Palestinians are often denied permits to travel there, even to receive desperately needed medical care (27).

The study was carried out in healthcare facilities: i.e., hospitals (governmental, non-governmental and private hospitals) with COVID-19 care units and COVID-19 healthcare centers. Healthcare professionals who were providing clinical care to patients were invited to participate in this study. The sample included medical doctors (specialized, residents, general physicians), nurses and nursing assistants, and allied health professionals (laboratory technicians, radiology technicians). A stratified purposive sample with probability proportional to size was used to select the healthcare facility and study participants. We selected the main governmental hospital, a private hospital with a COVID-19 care unit, and a COVID-19 healthcare center in each of the three study locations (i.e., Gaza Strip, the West Bank, and East Jerusalem). As a result, sixteen hospitals and medical centers were included in the study, out of a total of sixty.

### Data collection tool

This study questionnaire was a translated Arabic version by the study based on the data collection tool developed by the World Health Organization (WHO) in the protocol under the COVID-19 Research Roadmap (28). This study questionnaire was first translated into Arabic by the research team, and then back into English by a trained medical translator. Before piloting the questionnaire, the original English questionnaire and the back translated version were checked to ensure that the translation was accurate.

The study protocol was developed by experts in the Social Science and IPC Working Group who identified a pool of items based on WHO IPC interim guidance published in March 2020 (29, 30). We used a previous framework for studying clinician behavior, the Theoretical Domains Framework (TDF), in this study (30, 31). The TDF can promote the understanding of HCWs' behaviors, such as IPC practice, by examining potential underlying factors. It provides a framework that captures core constructs from multiple behavioral theories into 14 domains (32). Questions for this survey addressed the following TDF domains: knowledge; skills; social/professional role and identity; beliefs about capabilities; beliefs about consequences; environmental context and resources; and intentions, social influences, and emotions. Additional items in the survey, not included in the TDF framework, assessed three dimensions of institutional trust and were based on a previously validated measure (33). Therefore, TDF was applied in this study to identify determinants of HCWs' IPC behaviors during the COVID-19 pandemic to develop targeted strategies for optimizing such behaviors at this critical time (31–33).

To assess trust in health facilities and government, the survey tool included validated questions on HCWs responses regarding their trust in the institution where they worked and comprised the three different dimensions of institutional trust: perceptions of competence, honesty, and actions that are in the employees' best interests (3). The three trust measures questions were: the health facility where I work is ready to manage COVID-19; the health facility where I work is being honest with staff when managing COVID-19; and the health facility where I work would act in the interest of its staff when managing COVID-19. The six-point scale used was: “all of the time;” “most of the time;” “more than half of the time;” “less than half of the time;” “some of the time;” “at no time.” The trust score internal consistency coefficient was 0.76 (Cronbach's α).

In addition, the following TDF domains items were used to further interpret the data- on seven-point Likert scale-: emotions, service demand, environmental context and resources, skills and intentions, beliefs about capabilities and consequences, social influences/professional role, and wellbeing.

The Emotions item score was based on responses to questions regarding perceived personal risk and fear on the job (i.e., I am concerned about the risk to myself of becoming ill with COVID-19; I am concerned about the risk to my family related to COVID-19 as a result of my job role; I am afraid of looking after patients who are ill with COVID-19) (Cronbach's α: 0.68). The Service Demand item score reflected perceptions of whether the health system can handle current and future patient demands (i.e., I am confident that the healthcare service where I work can manage current patient demand related to COVID-19 and I am confident that the healthcare service where I work can continue to manage patient demand related to COVID-19 over the next 3 months) (Cronbach's α:0.80). The Environment item reflected the clarity of reporting measures of exposures, guidance materials, and ease of access to infection control practices (Cronbach's α: 0.67). The Skills and Intentions combined items score reflected training, confidence, and use of PPE (Cronbach's α: 0.82). The Beliefs item score was calculated from answers regarding their beliefs in the effectiveness of PPE and IPC procedures, and the amount of strain these procedures create (Cronbach's α: 0.84). The ability and motivation of HCWs to follow IPC precautions (28), and the social support of the community and medical staff, were also assessed (34). The WHO-5 wellbeing item scale—a validated and generic global rating scale to measure subjective wellbeing during the previous 2 weeks—was also included and staff emotions throughout the pandemic were investigated (35–37) (Cronbach's α: 0.86). The seven-point Likert scale ranged from “strongly disagree;” ‘Disagree,” “Somewhat disagree,” “Neither agree nor disagree;” “Somewhat agree,” “Agree;” and “strongly agree.” However, item questions related to PPE use and knowledge of recommended infection prevention and control procedures when providing direct medical care to suspected or confirmed COVID-19 cases included “Yes” and “No” answers only.

Information was collected on participants' characteristics (age, gender, marital status, having children or older adults at home), role and experience at work, their experience of caring for patients with suspected or confirmed COVID-19 infection, and their exposure to COVID-19. The translated Arabic version was piloted before its use to test for language clarity.

### Data collection

An online self-administered survey method was used for data collection. An electronic version of the questionnaire was sent to the selected participants. The United Nations Office for the Coordination of Humanitarian Affairs (UNOCHA) data collection for humanitarian use software “Kobo Toolbox” was used for data collection (38). Field coordinators contacted the targeted healthcare facility, obtained the full list of participants (email or WhatsApp) from the personnel departments for all HCWs. The questionnaire was then sent to all employees working in the targeted healthcare facility.

### Data analysis

For descriptive analysis, demographic characteristics are presented as frequencies and mean and standard deviation (mean ± SD) depending on variable types. For the variables whose answers were using the 7-point Likert scale, most of the variables were re-categorized into a 5-point Likert scale due to the small difference between “strongly agree” and “agree” answers,” “somewhat agree” and between “strongly disagree,” “somewhat disagree” and “disagree” “answers.” Since the data shows very low frequencies in the answers of “strongly disagree,” and “disagree,” and low frequency for the answers of “strongly agree,” and “agree,” we summed the scale into 5-point Likert scales (Supplementary Figure 1). However, again we re-categorize the 5-Likert points into a 3-point scale due to low frequencies to have significant results in the analysis.

For HCWs' emotional wellbeing, i.e., the five WHO-5 statements, the participants' responses were summarized into a total raw score and multiplied by 4 to produce an individual total score from 0 to 100, with the higher end of the scale representing the best possible wellbeing (35). The mean and standard deviation for the WHO-5 score was calculated. The emotions index was the sum of three questions.

The trust index was the sum of the three questions. The mean, median, and standard deviation were calculated. The median was used as a cutoff point (50%) since it is equivalent for a total score index of less than half of the time total trust.

The bivariate analysis took place of the WHO-5 score that comprised data on gender, marital status, place of residence, job role, medical specialty, place of work during COVID-19 outbreak, type of organization, working in more than one place, daily contact with patients, monthly income, and HCWs contact with a suspected/confirmed COVID-19 case. A *T*-test and one-way ANOVA *p*-value were calculated: a two-tailed *P-*value < 0.05 is considered statistically significant. The mean and standard deviation (SD) of trust variables were calculated to analyze the level of trust in a healthcare facility. For further analysis, we used a cutoff point of 50%. A χ2 test was used for comparisons of the various variables with a trust score cutoff point of −50%.

Further multivariable regression analyses were performed to explore independent associations between different domains of the TDF and behavioral/social factors while adjusting for confounding factors. Binary logistic regression model, forward stepwise (Wald) method, was used for controlling for participants age, gender, place of work, type of institution, job role, location of work, direct vs. indirect care for COVID-19 patients. All study predictive variables (i.e., emotions, service demand, environmental context and resources, skills and intentions, beliefs about capabilities and consequences, social influences/professional role, wellbeing, and most recent PPE use) were included in the model. All variables and outcomes were defined before final analyses. Adjusted odds ratio (aOR) and their 95% confidence interval (95% CI) are presented. All analyses were performed with Statistical Package for Social Sciences V.25.0.2 (SPSS, Chicago, Illinois, USA).

### Ethical issues

Permission was obtained from the Palestinian Ministry of Health to conduct the study. Al Quds research ethics committee approved the study. The study was also evaluated by the ethical review committee at the WHO office and approved before study funding. Written information about the purpose of the survey and how the data will be used was provided at the beginning of the questionnaire. Individual informed consent for participation in this study was obtained electronically by acceptance to fill in the study questionnaire.

## Results

### Demographic characteristics

A total of 1,200 HCWs were approached and 876 participated in the study, with a response rate of 73%.

Table 1 shows that 65.6% of study participants were male, young and 70% of them were from the West Bank. About 65% were working in public healthcare facilities, half were senior nurses and 22% were resident physicians. Around 61% of the HCWs were working in the acute care units; 70% reported being in contact with a suspected/confirmed COVID-19 case, and 52% were in daily contact with COVID-19 patients. A 31% were caring for older adults (>70 years). Of the study participants, 24% reported being diagnosed with COVID-19 and 58% reported COVID-like symptoms. However, only 81% reported being tested for COVID-19.

**Table 1:** Characteristics of study population.

**Age**	**Mean (±SD)**	**32 (**±**7.79) years**	
		**Count (*N*)**	***N* %**
Gender	Female	301	34.4%
	Male	574	65.6%
	Total	875	
Place of work during	West Bank	612	69.9
COVID19 outbreak	Jerusalem	91	10.4
	Gaza Strip	173	19.7
	Total	876	
Ever diagnosed with	Yes	207	23.6%
COVID-19	No	669	76.4%
	Total	876	
Ever been tested for	Yes	704	80.8%
COVID-19?	No	167	19.8%
	Total	871	
Job role^†^	Senior nurse	448	51.1%
	Assistant nurse	59	6.7%
	Specialized doctor	79	9.0%
	Resident doctor	195	22.3%
	Allied health profession	80	9.1%
	Others	14	1.6%
	Total	875	
Medical specialty ^†^^†^	Acute care	513	61.3%
	Internal medicine	80	9.6%
	Surgery	51	6.1%
	Pediatrics	34	4.0%
	Others	159	19.0%
	Total	837	
Type of organization	Governmental	572	65.4%
	Non- governmental	303	34.6%
	Total	875	
HCWs contact with a	No	101	12.1%
suspected/confirmed	Yes	732	87.9%
COVID-19 case	Total	833	

^†^^†^others: Laboratory, maternity departments, general clinics, neonate department,

^†^Acute care (anesthesiology, ER, ICU, infectious disease unit). SD: standard deviation.

Healthcare systems were forced to adapt to the pandemic. About 85% of healthcare facilities closed key departments and transformed them to offer COVID-19 care provision; 90% of the facilities targeted had dedicated sections. In addition, 50% of HCWs reported being transferred from their departments to COVID-19 departments.

### Wellbeing of participants

In our study, the mean score of the WHO-5 wellbeing score was 35.96 (SD: 21.8) with a median of 36.0. Males showed significantly lower psychological wellbeing mean score values (34.8, SD 21.3) than females (38.2, SD 22.56) (*p* < 0.05), as did HCWs working with COVID-19 patients (34.5, SD 20.9) compared with those non-working with them (39.4, SD 23.3) (*p* < 0.05) (see Supplementary Table 1). Using a cutoff point of 50%, 76% of HCWs had poorer wellbeing during the COVID-19 pandemic. Finally, we conducted multivariable logistic regression to assess the influence of various participant characteristics on HCWs' wellbeing; none of these characteristics predicted the WHO-5 wellbeing cutoff point of 50%.

### Healthcare workers emotions and sense of control during the pandemic

In our study, 51% of HCWs reported that getting infected with COVID-19 was out of their control, but 80% agreed that this risk was part of their job. Regarding concerns about exposure to COVID-19, while caring for patients, about half (45%) of HCWs reported fear, with 90% of them worried to transfer the infection to their families and 75% concerned to contract the illness themselves. In the multivariate ordinal logistic regression analysis to assess the influence of participants' characteristics on HCWs emotions and sense of control, none of the participants' characteristics predicted emotions or sense of control.

### Protection, training, and PPE availability at work

**Figure 1:**
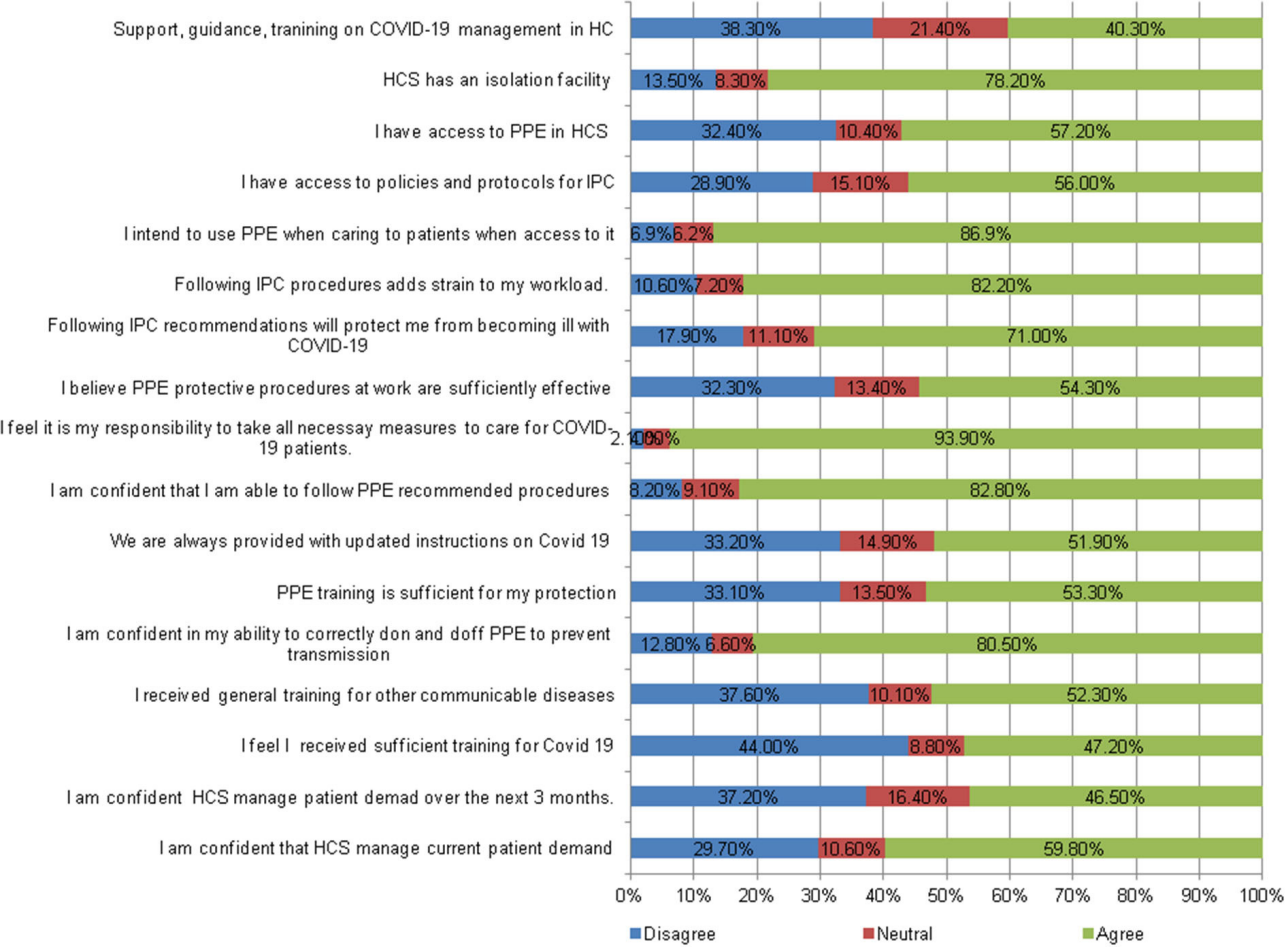
Perceptions of healthcare workers on recommended IPC use intentions, skills, belief in consequences and capabilities, social role, and environmental resources.

In our study, 78% of HCW reported that there was an isolation unit in their healthcare facility. However, only 40% reported receiving support, guidance, or training on COVID-19 management in the healthcare facility; 50% reported access to policies and protocols of prevention and control of COVID-19 (Figure 1).

On the availability and use of IPC, 87% reported their intention to use PPE when caring for patients, although 57% reported having access to PPE in their healthcare facility. Also, 52% reported being provided with updated instructions about COVID-19, and half reported receiving sufficient training on the use of PPE. Around 80% of HCWs reported confidence in their ability to use PPE properly to protect themselves and prevent transmission of infection, although 53% felt that they did not receive proper training in protection (Figure 1).

Health care workers reported that 41% of their sources of infection prevention information in the previous 2 weeks were social media, 24% were hospital training, 22% were official government websites, 2% were family and friends, and 11% came from other sources.

HCWs had a moderate belief level (50%) that it is their responsibility to take protective measures to protect themselves while caring for COVID-19 patients and 50% of them believed that using PPE would protect them sufficiently from becoming infected at work. Also, 82% of HCWs believed that following the recommended procedures for the control of COVID-19 added a significant strain to their workload (Figure 1).

### Trust in institution

**Figure 2:**
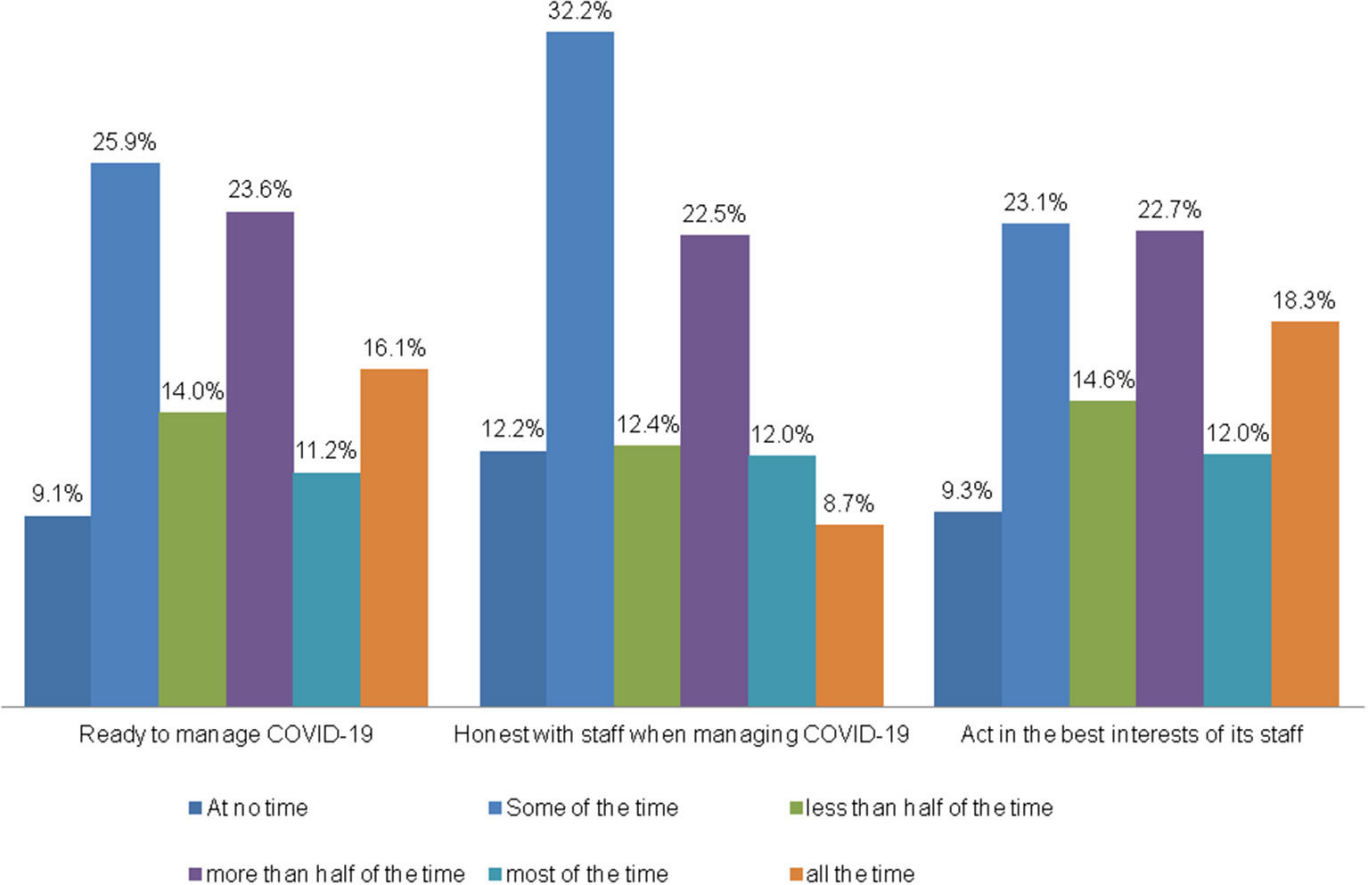
HCWs' trust in the health care facility while managing COVID-19 pandemic.

In the study, HCWs were asked about their trust in the healthcare facility in managing COVID-19, being honest with staff, and acting in the best interests of staff. The mean trust score was 7.73 (standard deviation 3.86) and the median was 8.0 (range 0–15). During the pandemic, 50.9% of HCWs believed their organization could manage the healthcare facility (more than half of the time). Also, 43% trusted that their healthcare facility would be honest with staff (more than half of the time and more), and 53% trusted it can act in the best interests of staff (Figure 2). The mean of the three variables that represent participants' answers on institutional trust was 7.72 (SD 3.86) and the median was 8.0. This represents moderate trust by HCWs in their healthcare facility.

HCWs living in cities showed the highest mean in trust compared with participants living in other areas, and those working in East Jerusalem hospitals compared with workers in the West Bank and Gaza Strip (*p* < 0.05). Other variables did not indicate any significant difference. Using the cutoff point of 50%, 535 participants (49.7%) showed high trust in their organization. When comparing trust at the cutoff of 50%, only the place of residence and type of organization showed a significant difference in *p*-value 0.05 (Table 2).

**Table 2:** Trust with cutoff point 50% in comparison with study variables.

		**Less than 50%**		**More than 50%**		
		**Count**	**%**	**Count**	**%**	**χ2 test**
						***p*-value**
Gender	Female	139	32.0%	162	36.8%	0.13
	Male	296	68.0%	278	63.2%	
	Total	435		440		
Job role	Senior nurse	26	6.0%	33	7.5%	0.088
	Assistant nurse	223	51.4%	225	51.0%	
	Specialized doctor	37	8.5%	42	9.5%	
	Resident doctor	110	25.3%	85	19.3%	
	Allied health profession	30	6.9%	50	11.3%	
	others	8	1.8%	6	1.4%	
	Total	434		441		
Medical specialty article	acute care	245	58.8%	268	63.8%	0.193
	Internal medicine	49	11.8%	31	7.4%	
	surgery	23	5.5%	28	6.7%	
	Pediatrics	19	4.6%	15	3.6%	
	others	81	19.4%	78	18.6%	
	Total	417		420		
Place of work during COVID-19 outbreak	West Bank	**327**	**75.2%**	285	64.6%	**0.002**
	Jerusalem	33	7.6%	58	13.2%	
	Gaza	75	17.2%	98	22.2%	
	Total	435		441		
Type of organization you are working with	Governmental	297	68.4%	274	62.1%	0.050
	Non- governmental	137	31.6%	167	37.9%	
	Total	434		441		
Direct contact with COVID-19 patients	No	138	31.7%	128	29.0%	0.39
	Yes	297	68.3%	313	71.0%	
	Total	435		441		

Bold values are significant p-values.

### Multivariate analysis

In Table 3, the bivariate logistic regression model showed the factors that determine HCWs' institutional trust as reflected in the responses about whether HCWs believed that their health facility was competent, honest, and acted in the best interests of its staff. The model shows that confidence in the system's ability to manage COVID-19 cases, encouraged and supported by senior medical/nursing staff to apply recommended infection prevention and control measures, increases trust in the organization. In addition, the wellbeing of HCWs was linked to greater trust in institutions.

**Table 3:** Binary logistic regression for the association of perceived skills, self-reported environmental context, social influences, emotions, recent use of IPC, emotions index and wellbeing with institutional trust.

		**Trust**				**Crude odds ratio**				**Adjusted odds ratio**			
		**Less than 50**		**More than 50**		**Sig**.	**OR**	**95% CI OR**		**Sig**.	**aOR**	**95% CI aOR**	
		***N*** = **435**		***N*** = **441**									
		***N***	**%**	***N***	**%**			**L**	**U**			**L**	**U**
I am confident that the healthcare service where I work can continue to manage patient demand related to COVID-19 over the next 3 months.	Disagree	182	41.9	76	17.4		1.00				1.00		
	Neutral	54	12.4	38	8.7	000	1.68	1.03	2.67	0.004	2.02	1.25	3.28
	Agree	198	45.6	322	73.9	000	3.48	2.56	4.73	0.025	1.56	1.06	2.30
I have received general training for infection, prevention and control procedures for other communicable diseases	Disagree	210	48.4	119	27.0		1.00				1.00		
	Neutral	51	11.8	37	8.4	0.31	1.28	0.79	2.06	0.383	0.782	0.450	1.36
	Agree	173	39.9	285	64.6	000	2.80	2.17	3.90	0.051	1.447	1.00	2.10
I intend to always use the recommended PPE when taking care of patients with suspected or confirmed COVID-19 when I have access to these.	Disagree	29	6.7	31	7.1		1.00				1.00		
	Neutral	35	8.1	19	4.3	0.07	0.51	0.23	1.06	0.017	0.320	0.126	0.814
	Agree	370	85.3	389	88.6	0.95	0.98	0.58	1.66	0.009	0.42	0.22	0.809
In the health facility where I work, I have access to clear policies and protocols for everyone to follow related to infection prevention and control procedures for COVID-19	Disagree	183	42.3	69	15.7		1.00				1.00		
	Neutral	78	18.0	54	12.3	0.007	1.84	1.18	2.86	0.12	1.51	0.89	2.55
	Agree	172	39.7	317	72.0	000	4.89	3.50	6.82	0.000	2.631	1.703	4.06
I am encouraged and supported by senior medical/nurse staff to apply recommended infection prevention and control measures	Disagree	131	30.3	49	11.1		1.00				1.00		
	Neutral	94	21.7	58	13.2	0.034	1.65	1.04	2.62	0.288	1.341	0.780	2.30
	Agree	208	48.0	334	75.7		4.29	2.96	6.22	0.002	2.03	1.29	3.20
Emotions index	<50	45	10.3	82	18.6		1.00				1.00		
	≥50	390	89.7	359	81.4	0.001	0.51	0.34	0.75	0.034	0.596	0.37	0.961
WHO-5 wellbeing	<50	351	80.7	315	71.4		1.00				1.00		
	≥50	84	19.3	126	28.6	0.001	1.67	1.22	2.29	0.032	1.52	1.04	2.22
PPE availability during last clinical shift^†^	Mean ± SD	5.47 ± 2.08	6.55 ± 1.71	000	1.37	1.26	1.47	0.001	1.18	1.07	1.29

aOR, adjusted odds ratio; COVID-19, coronavirus disease 2019; CI, confidence interval. Binary logistic regression model after controlling for age, gender, place of work, type of institution, job role, location of work, direct vs. indirect care for COVID-19 patients.

^†^PPE availability in the last week is the index sum of availability of: Hand soap, N95 respirator (FFP1 or equivalent), surgical mask, disposable apron, fluid-resistant gown, eye protection (i.e., goggles or face shield, and gloves). SD, Standard Deviation; L, lower; U, Upper.

Proper training on prevention and control procedures for other communicable diseases; access to clear policies and protocols for everyone to follow related to infection prevention and control procedures for COVID-19; and the PPE availability during the previous clinical shift also increased trust in the organization during the pandemic. However, HCWs reported that when having access to recommended PPE, the intention to use it to care for patients with suspected or confirmed COVID-19 was inversely associated with trust. This was like the emotions index, i.e., staff concerned about becoming sick due to the risk of self-exposure and infecting their families.

## Discussion

This is the first study in Palestine that provides insight into the perceptions of HCWs and the barriers and facilitators that influence the trust of staff in the institutions where they work.

This trust ultimately shapes adherence to prevention and control measures during the COVID-19 pandemic and organizational resilience. In general, the findings showed that HCWs have moderate levels of trust in their institution to manage the healthcare facility during the pandemic; be honest with staff, and act in the best interests of their staff. Several work- related factors associated with institutional trust (IT) were investigated in this study. Some personal factors like the HCWs' job role, their medical specialty, location of work, and working in high-risk units did not show a significant relationship with IT. However, confidence in the system's ability to manage COVID-19 cases and encouragement and support from senior medical/nursing staff to apply recommended infection prevention and control measures increase trust in an organization. Other factors related to IT during a pandemic include receiving proper training on prevention and control procedures for other communicable diseases, having access to clear policies and protocols for everyone to follow related to infection prevention and control procedures for COVID-19, and the availability of PPE during the previous clinical shift.

One of the key findings of this study is that wellbeing of HCWs is associated with IT. Greater trust was reported by those with good mental health like being cheerful, relaxed, sleeping well, and feeling active. However, those worried about themselves or their families being infected with COVID-19 showed lower trust in the institution. Similar findings have been reported worldwide. Psychological strain among HCWs in European hospitals was shown to be high; one-third of HCWs reported fear in dealing with COVID-19 patients, and almost all respondents were worried about the risk to their families due to their job (mean 56.3, SD ± 19.3) (38). In our study, the situation of Palestinian HCWs was shown to be worse than that of HCWs in Europe (39). The mean of the WHO-5 wellbeing scores was 35.96 (SD ± 21.8), which was significantly higher among female HCWs than males (38.2 vs. 34.8), and 75% of the participants reported a poor wellbeing index. A high level of fear was reported by 50 percent of health professionals in Gaza who had never worked with COVID-19 patients before compared with 27.6% who had work experience with COVID-19 patients (40). Among Saudi Arabian HCWs, 27.1% scored high on a negative emotional impact scale (41); in Germany, the COVID-19 pandemic had a negative impact on HCWs mood (48.3%), as well as restricted their private lives (42). Zhang and colleagues reported similar results in China, showing a high prevalence of severe insomnia, anxiety, depression, somatization, and obsessive-compulsive symptoms (43). In this study, multivariate analysis showed that a good wellbeing is associated positively with trust in the organization (adjusted OR 1.52, 95% CI 1.038–2.22).

In this context, the psychological distress experienced by healthcare workers may be related to their concerns about safety at work (7) and their lack of understanding of the virus. HCWs may also be worried about the shortage of medical protective equipment, the long-term workload, and the lack of rest. The study highlights that trust in an institution may be boosted by providing proper training and essential medical materials and equipment. It should also provide the proper protection and preventive measures for its employees; improve communication, establish clear protocols, and provide PPE that could enhance trust and, thus, employees' psychological wellbeing.

The TDF scale (31) was applied in this study to understand IPC behaviors during the COVID-19 pandemic and to develop targeted strategies for optimizing such behaviors at this critical time.

One key finding in this study was that HCWs lacked a sense of control during the pandemic period. Becoming infected with COVID-19 was perceived to be out of their control, although 80% agreed that this risk was part of their job. Half of HCWs (50%) felt fear when caring for COVID-19 patients; feared becoming infected while caring for patients with COVID-19 (75%) and feared transferring the infection to their families (90%). These findings indicate a high level of fear and stress among Palestinian healthcare workers during the COVID-19 pandemic. Maraqa et al. study (2020) showed that 74.0% of Palestinian HCWs reported high-stress levels during the outbreak. Fear of spreading the infection to family members was the main source of stress (91.6%) (44). Comparable results were seen in Germany where most HCWs described moderate concerns about their health (41.9%) but had strong concerns about the health of others (46.0%) (41). A study in Saudi Arabia during the Middle East respiratory syndrome coronavirus (MERS) pandemic, showed that more than two-thirds of HCWs were worried about being infected through exposure to infected patients. It reported that the most frequently reported reasons for worry were the ability of the virus to cause severe disease or death and lack of a specific treatment (37). A hospital-based study during the MERS outbreak showed that many health workers worried about becoming sick and possibly infecting others (45).

Another important finding in this study is the lack of awareness by HCWs about prevention measures and their use; this may be one of the major reasons for the feeling of loss of control. A national Palestinian study showed that HCWs surveyed did not receive adequate training on local protocols or measures to address COVID-19 spread (58.7%) (46). In Cyprus, a study indicated that poor knowledge regarding preventive measures may directly increase the risk of COVID-19 spread (47). In addition, lack of means of protection, poor training, and inadequate PPE availability at work were strong factors affecting fear and loss of control among HCWs. In the study multivariate model, determinants for IT were receiving general training in IPC procedures for other communicable diseases, alongside access to clear policies and protocols for everyone to follow related to infection prevention and control of COVID-19. These results were like a German study in which 47.2% of all participants reported that their employer had provided specific COVID-19 training during the pandemic, and that this training was provided more often to doctors (50.9%) than to nursing staff (39.3%) (47). HCWs who received PPE training in the previous 2 years reported using the most elements of PPE and more frequently than those who did not report PPE training (48). On the contrary, in Saudi Arabia, 95.5% of HCWs reported receiving training on the safe use of personal protective tools (35). In focus group discussions in the United States, inadequate access to COVID-19 testing and uncertainty about whether their organization would support their needs if they developed an infection, was among several other factors that caused HCWs anxiety and could undermine their trust in their organizations (5). The early implementation of PPE training should be a requirement to reduce the spread of COVID-19 among HCWs (48). PPE training specifically for COVID-19 would have the most significant impact on the proper use of PPE and thus, on staff concerns and trust in their institutions.

Although 87% of HCWs reported their intention to use PPE when caring for patients, a low percentage (57%) reported having access to PPE in their healthcare facility in the current study. In the multivariate model, the intention to use PPE while caring for suspected or confirmed cases was inversely associated with IT. Interestingly, in this study, 50% of HCWs believed that using PPE would protect them sufficiently against becoming infected at work. Globally, the availability of PPE is higher in some countries than in others. In Cyprus for example, 38.7% of HCWs believed that adequate and appropriate protective equipment was readily available (47). In Germany, over 40% of medical professionals stated that there was a regular (18.1%) or even permanent (16.5%) shortage of equipment at their institution (42). In Palestine, HCWs reported lacking in hand sanitizer (51.4%), gloves (48.6%), facemasks (72.5%), eye protection (goggles/glasses: 92.8%), and face shields (92.0%) (46). Institutional trust is a key risk attenuator for HCWs to adhere to recommended IPC use. In Saudi Arabia, the presence of a hospital policy to address employees with suspected or known exposure to the COVID-19 virus and the implementation of preventive measures reduced the negative emotional response between HCWs (41).

During pandemics, HCWs trust improves when they empowered and supported by their managers. When HCWs feel psychologically safe, this enables better patient safety in everyday practice for all patients (5, 6). In the study multivariate model, having confidence in the system's management of COVID-19 cases, alongside encouragement and support from senior medical/nursing staff to apply the recommended infection prevention and control measures were strong determinants for institutional trust. The absence of managerial support for emotional distress can be detrimental to trust and the psychological safety of HCWs. Therefore, managers need to support HCWs and deal with any signs of emotional distress during COVID-19 (21).

Sources of information globally have been very much dependent on social media and internet access. In this study, HCWs reported that social media was their main source of information about COVID-19 (41%), followed by hospital training (24%), and official government websites (22%). In Saudi Arabia, the main source of information about the Middle East respiratory syndrome (MERS) was the internet (26%) (48). In Canada, social media was reported as a primary source of information, and healthcare workers were not satisfied with the information provided by institutions on COVID-19 (49). However, a study by Al-Ashwal et al. in Yemen found that television and radio were the main sources of information (69.5%), followed by social media (63.6%), and only 25.5% of HCWs acquired knowledge from peer-reviewed scientific articles (50). Staff may seek information *via* social media because of the high risk of infection posed by the COVID-19 virus that prompts HCWs to gain a better understanding of the nature of the disease, the characteristics of the causative agent, evaluation of self-susceptibility and vulnerability, and to evaluate the efficacy of the available preventive measures (34). Another possible explanation is that this disease is new and health institutions were not well-prepared to face this challenge due to a lack of scientific information about it. This could motivate HCWs to search social media for the latest information.

Our study had some limitations. The survey took place during the second peak of the pandemic and under a partial lockdown. In this period, HCWs experienced extreme stress at work and at their personal level which may exaggerate their responses. Also, this is a cross-sectional study which makes it a challenge to identify the cause–effect relationship between the independent and dependent variables. In addition, obtaining the data through self-report questionnaires makes it liable for reporting bias; those interested in the topic of feeling stress chose to respond. Also, we were unable to compare the differences between responders and non-responders.

Despite the caution in the generalization of the findings, the findings of the current study about HCWs' trust in their organization are crucial contribution to the literature review.

### Practical implications

The study has practical implications for crisis communication and management. Its findings can be tailored to provide a set of recommendations that can be used to limit the negative outcomes associated with low levels of trust in institutions during health crises like the COVID-19 pandemic in the Palestinian context.

Changing infrastructure, work policies, and staffing to reduce risk and weariness in order to adjust service delivery in such pandemics is necessary. Capacity building across all cadres for emergency preparedness should be fostered to ensure a smooth transition of HCWs from diverse divisions/specialties to emergency response circumstances. In collaboration with the WHO, the Ministry of Health and other healthcare providers must conduct systematic and periodic training on IPC protection protocols. Training protocols must be continually updated and distributed to HCWs *via* tele-health systems, organizations' websites, and personal e-mails. Therefore, digital triaging could be used as a less resource-intensive way to protect HCWs from emerging viral infections, which can be done through structural changes in health facilities to easy triaging. Moreover, illness surveillance methods and health information infrastructures must be strengthened to have data analytics in health surveillance.

Additionally, the institutions should facilitate access to mental health resources such as psychological counseling, practicing meditation, and debriefing. For example, developing HCWs community groups that allow connections and reduce feelings of isolation would help in socializing within these teams.

Increased human resources, training response teams, and providing housing for teams to be away from their families and alleviate stress should all be part of the disaster preparedness plan. Also, the institutions should also provide individual and organizational support to HCWs in nutrition, physical exercise, sleep quality, and reducing burnout. Furthermore, communication with leadership should be improved to facilitate problem solutions and provide incentives (such as specific raises in salary and personal recognition) to encourage HCWs motivation. In addition, health institutions should work to improve human resources and support supplies to reduce workload.

### Policy implications

The main findings of our study show that several factors, including crisis management, policy decision-makers' wellbeing, and health professionals' physical and emotional wellbeing, might influence employee trust in institutions. As a result, local policy guidelines must be developed in collaboration with various healthcare providers and implemented in future outbreaks.

A policy for enhancing working conditions in terms of employment stability and social security should be implemented. This might be accomplished by enhancing HCWs' terms and conditions of employment by altering their pay scale, which could be secured by locating suitable financing sources. Furthermore, policies are required to foster a working environment that protects HCWs' mental health and wellbeing, thereby improving their quality of life and achieving a better work-life balance. In such instances, sustaining the provision of services by various healthcare providers, particularly the Ministry of Health, to enable the procurement of products and services, ensure the supply of medicines, and ensure the supply of personal protective equipment (PPE), is also critical. These policies, which address the protection and care of HCWs, indicate the need for more investment in this area.

Based on the COVID-19 pandemic experience, the MoH should have an emergency preparedness plan. To effectively deliver best practices, the plan should provide training and essential medical materials and equipment, including management based on the latest evidence and provision of appropriate protection and prevention measures. Therefore, a task force maybe created to help mitigate physical, mental, social or economic effects on HCWs, even after the current pandemic is over.

The research institutions and universities need to carry out studies to understand the effect of communication strategies such as media impact and information sharing on workers' perspectives. Also, understanding the environmental influences such as social and cultural beliefs will assist in developing potential interventions to support HCWs in future pandemics.

## Conclusions

In general, the findings showed that HCWs have moderate levels of trust in their institution to manage the healthcare facility during the pandemic; be honest with staff, and act in the best interests of their staff. Several factors were associated with institutional trust (IT) such as receiving proper training, having access to clear policies and protocols, the availability of PPE, and feeling emotional. Therefore, strategies to promote trust and resilience in healthcare workers must be developed and implemented to counter the psychological distress they faced during this crisis. HCWs should be provided with clear, accessible communications about policies and protocols, as well as training about infection prevention and control, personal protective equipment, and support during pandemics to increase their trust in the healthcare system. Additionally, the improvement in HCWs' wellbeing can be attributed to a greater sense of trust in institutions. Finally, policymakers and authorities should invest in training and better employment circumstances for HCWs to ensure long-term healthcare security in reaction to the COVID-19 pandemic or possible future epidemics.

Future work is needed for evaluating factors that contribute to change in trust, beliefs, and skills during outbreaks, in addition, to determining the proper policies needed to be implemented in these healthcare settings.

## Author's note

Nuha El Sharif is an associate professor of Public Health. Muna Ahmead has a PhD in Mental Health. Asma Imam is an associate professor of Health Management and Quality Control. El Sharif has research experience in healthcare workers' exposure in the workplace, cancer epidemiology and other non-communicable diseases, and extensive experience with data analysis and model development. Ahmead has experience in research related to PTSD, cancer, depression, fear of death, quality of life, and other mental health issues. Imam's main research interests are in quality of life with emphasis on cancer patients and the elderly, and quality of healthcare and reproductive health.

## Data availability statement

The original contributions presented in the study are included in the article/Supplementary material, further inquiries can be directed to the corresponding author/s.

## Ethics statement

The studies involving human participants were reviewed and approved by Palestinian Ministry of Health Ethical Committee (REF: R0/1508/11/59) and Al Quds University Research Ethical Committee (Ref No. 150/Rec/2020) in accordance with the Declaration of Helsinki. The patients/participants provided their written informed consent to participate in this study.

## Author contributions

NS and AI designed the survey and developed the study tool. NS was responsible for supervision of software development, data collection, data entry, and study analysis. NS, MA, and AI participated and were responsible for writing the manuscript. All authors read and approved the final manuscript.

## Funding

The study was funded by the World Health Organization, Geneva, Switzerland (Grant number 2020/1059265-2).

## Conflict of interest

The authors declare that the research was conducted in the absence of any commercial or financial relationships that could be construed as a potential conflict of interest.

## Publisher's note

All claims expressed in this article are solely those of the authors and do not necessarily represent those of their affiliated organizations, or those of the publisher, the editors and the reviewers. Any product that may be evaluated in this article, or claim that may be made by its manufacturer, is not guaranteed or endorsed by the publisher.

## Abbreviations

CI: confidence intervals
COVID-19: coronavirus disease 2019
HCWs: healthcare workers
IPC: infection prevention and control
MERS: middle east respiratory syndrome
MOH: ministry of health
PPE: personal protective equipment
OR: odds ratio
TDF: theoretical domains framework
UNOCHA: coordination of humanitarian affairs
WHO: world health organization.
