# Supplementary figures and images for: COVID-19 infection prevention and control procedures and institutional trust: Perceptions of Palestinian healthcare workers
Source: Front Public Health. 2022 Aug 19;10:947593. doi: 10.3389/fpubh.2022.947593 (PMC9437519; doi:10.3389/fpubh.2022.947593)

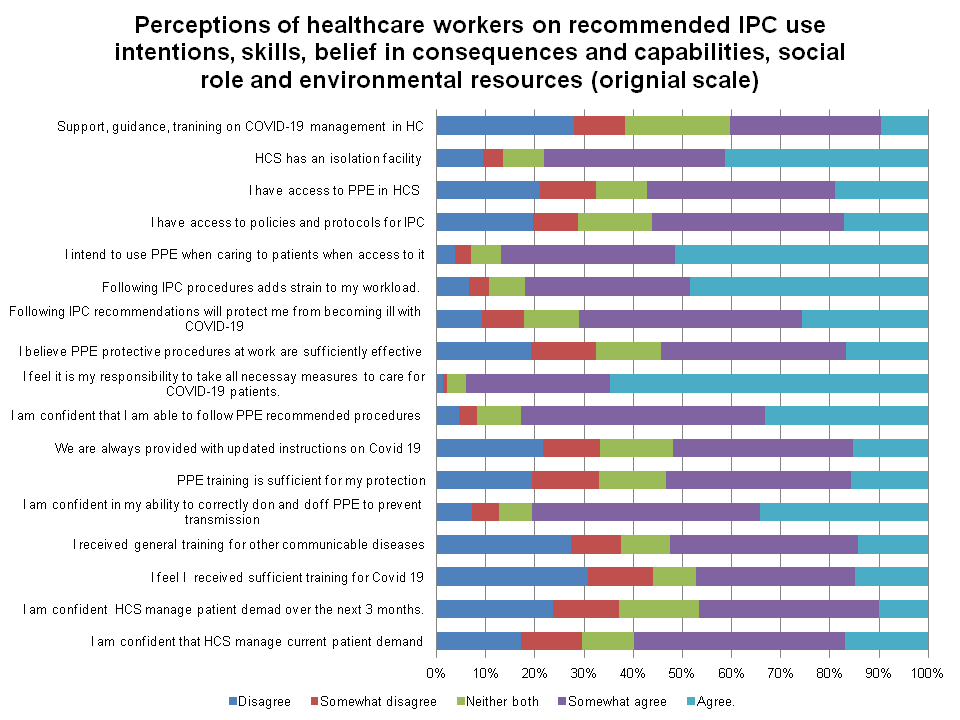

Supplement: Supplementary file 1 [file Image_1.tif]
