# Supplementary material for: COVID-19 infection prevention and control procedures and institutional trust: Perceptions of Palestinian healthcare workers
Source: Front Public Health. 2022 Aug 19;10:947593. doi: 10.3389/fpubh.2022.947593 (PMC9437519; doi:10.3389/fpubh.2022.947593)
Supplement: Supplementary file 2 [file Table_1.docx]

Table S1: WHO-5 Emotional Well-Being index per subgroup

|  | | Mean | Standard Deviation | T test  p value |
| --- | --- | --- | --- | --- |
| Gender | Female | 38.18 | 22.56 | 0.03 |
|  | Male | 34.82 | 21.28 |  |
| Place of residence | Village | 35.01 | 21.72 | 0.60 |
|  | City | 36.79 | 21.64 |  |
|  | Camp | 36.38 | 22.67 |  |
| Place of work during COVID19 outbreak | West Bank | 35.76 | 22.25 | 0.36 |
|  | Jerusalem | 34.42 | 16.26 |  |
|  | Gaza | 37.50 | 22.58 |  |
| Type of organization you are working with | Governmental | 35.14 | 22.30 | 0.11 |
|  | Non- Governmental | 37.61 | 20.64 |  |
| Job role | Senior nurse | 38.2 | 23.0 | 0.38 |
|  | Assistant nurse | 35.6 | 21.9 |  |
|  | Specialized doctor | 39.4 | 21.1 |  |
|  | Resident doctor | 33.7 | 22.5 |  |
|  | Allied health profession | 37.8 | 19.5 |  |
|  | others | 37.1 | 16.9 |  |
| Medical specialty * | acute care | 35.4 | 21.3 | 0.37 |
|  | Internal medicine | 34.6 | 23.6 |  |
|  | surgery | 39.4 | 22.5 |  |
|  | Pediatrics | 42.6 | 19.7 |  |
|  | others | 36.7 | 22.8 |  |
| HCWs contact with a suspected/confirmed COVID-19 case | No | 39.4 | 23.3 | 0.02 |
|  | Yes | 34.5 | 20.9 |  |

COVID-19, corona-virus disease 2019; HCW, health care worker; NS, not significant; SD, standard deviation; WHO, World Health Organization
